# Supplementary material for: Evaluation of the Antimicrobial Effect of Graphene Oxide Fiber on Fish Bacteria for Application in Aquaculture Systems
Source: Materials (Basel). 2022 Jan 26;15(3):966. doi: 10.3390/ma15030966 (PMC8840572; doi:10.3390/ma15030966)
Supplement: Supplementary file 1 [file materials-15-00966-s001.zip › materials-1526868-supplementary.pdf]

materials

Supplementary Material

Evaluation of the Antimicrobial Effect of Graphene Oxide Fiber on Fish Bacteria for Application in Aquaculture Systems

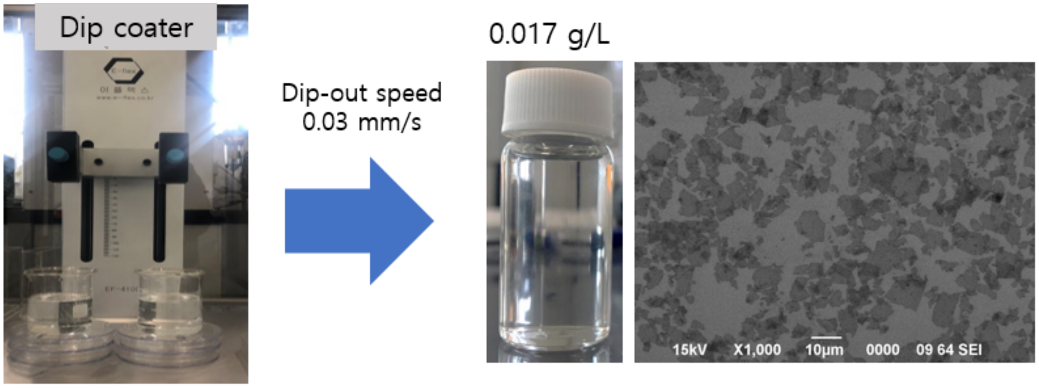

Figure S1. SEM sample preparation of graphene oxide using Dip coater.

Table S1. Metallic impurities contents in graaphene oxide as determined by ICP-MS analysis.

Metal	Unit	Result
Al (Aluminum)	mg/L, ppm	41.1
Fe (Iron)		379
K (Potassium)		114
Mn (Manganese)		65.5
Na (Sodium)		309
Si (Silicon)		124
